# Nutrition-Based Paternal Influence on Gynecological Diseases in Female Offspring via Epigenetic Mechanisms

**DOI:** 10.3390/nu17233690

**Published:** 2025-11-25

**Authors:** Titilayomi J. Durojaye, Sebanti Ganguly, Yuanyuan Li, Trygve O. Tollefsbol

**Affiliations:** 1 Department of Biology, University of Alabama at Birmingham, Birmingham, AL 35294, USA; jtduroja@uab.edu (T.J.D.); sebantig@uab.edu (S.G.); 2 Department of Nutrition and Food Science, University of Maryland, College Park, MD 20742, USA; roseli15@umd.edu; 3 O’Neal Comprehensive Cancer Research, University of Alabama at Birmingham, Birmingham, AL 35294, USA; 4 Nutrition Obesity Research Center, University of Alabama at Birmingham, Birmingham, AL 35294, USA; 5 Comprehensive Diabetes Center, University of Alabama at Birmingham, Birmingham, AL 35294, USA

**Keywords:** gynecology, nutrition, epigenetic, sperm, phytochemical, ovarian cancer, breast cancer

## Abstract

Studies have widely indicated that the composition of maternal nutrition and diets might affect offspring health later in life. Studies on paternal contribution to the offspring’s disease are relatively scarce but are an important subject to the field. Recent research has suggested that paternal factors influenced by nutrition have been implicated in the transgenerational heritage of health and diseases through epigenetic mechanisms. This review aims to explore the current state of knowledge on nutrition-based paternal impacts on gynecological disease through epigenetics, focusing on the transmission of cancer and metabolic diseases from father to female offspring. We will explore the various mechanisms by which epigenetic landmarks, such as DNA methylation, histone modifications, and non-coding RNAs, are passed on through sperm and reprogrammed in the embryo, influencing offspring development and health. We will discuss the impacts of preconception paternal nutrition on two common cancer such as breast cancer and ovarian cancer in female offspring. Additionally, paternal overweight or obesity has been associated with increased risk of obesity in the offspring and compromised metabolic health, which may link to reproductive conditions such as infertility. Understanding the molecular mechanisms underlying non-genetic inheritance is crucial for elucidating the nutrition-mediated developmental origins of health and disease. This review highlights the mechanistic correlation between preconception paternal nutrition and female offspring gynecological health. Furthermore, it emphasizes the need for additional research to establish evidence-based paternal nutrition consultation and guidelines aimed at optimizing reproductive health and pregnancy outcomes in couples planning to conceive.

## 1. Introduction

Epigenetics has a compounded role in cancer, affecting different facets of tumor biology [1,2]. Processes such as histone modification, DNA methylation, nucleosome remodeling, and non-coding RNA expression play pivotal roles in many biological mechanisms essential for cancer development that alter gene expression without changing the DNA sequence itself [3,4]. Also, epigenetic mechanisms are key in modulating cell cycle and steering gene expression which are important factors in cancer development [5]. Epigenetic alterations contribute to the onward motion and spread of cancer by influencing several cellular functions [6]. For instance, they can impact how pathogens recognize receptors or lead to mis-regulation of imprinted genes which promote tumor development by allowing uncontrolled cell growth [6,7]. Ovarian cancer is among the most fatal and common gynecological malignancies. It is distinguished by challenges in early diagnosis, high recurrence rates, and resistance to existing treatment [8,9]. The tumor microenvironment plays a vital role in its growth and metastasis, with peritoneal metastasis being facilitated by the interaction between tumor cells and other cells such as tumor-associated macrophages [10,11]. Hormonal factors, specifically estrogens, are implicated in its development and play a role in its tumor biology [12]. Recent advancements in therapies include targeting these macrophages and employing immunotherapy and ferroptosis, a form of programmed cell death showing promise in suppressing tumor growth [13,14]. Breast cancer is a prime global health issue and the most common cause of cancer deaths among women [15]. Many risk factors contribute to its development including age, genetics, family history, lifestyle, and hormonal factors [15,16]. Breast cancer shows a complex molecular biology with signaling pathways like ER, HER2, and Wnt playing essential roles in its progression [17]. Gene mutations such as BRCA1 and BRCA2 significantly elevate risk levels, highlighting the need for targeted genetic therapies [17].

The outstanding nature of nutrition-mediated paternal epigenetic impact on the health and development of offspring is becoming extensively acknowledged and is now seen as a critical element in transgenerational inheritance [18,19,20]. Environmental exposure, lifestyle factors such as diet, stress, toxicants, and father’s age can affect the epigenome of sperm cells [21,22,23] which can then influence the development of offspring through epigenetic mechanisms, like histone modification, DNA methylation, and non-coding RNAs [24,25,26]. Importantly, paternal nutritional choices in the diet have been shown to have effects on sperm which can affect the health of the offspring [27].

Traditionally, research has focused on maternal influence on offspring, but recent evidence indicates that paternal factors also substantially alter offspring traits through epigenetic processes [18,19]. This review focuses on the role of paternal dietary factors in influencing breast and ovarian cancer risk in offspring through epigenetic mechanisms. We explore how paternal intake of macro- and micronutrients, as well as bioactive compounds, can induce epigenetic changes in sperm DNA methylation, histone modifications, and non-coding RNAs. By synthesizing current evidence and identifying knowledge gaps, we aim to stimulate further investigation into the transgenerational effects of paternal diet on female cancer risk in offspring and potential interventions to lower these risks.

## 2. Mechanisms of Paternal Epigenetics and Its Role in Gynecological Cancers

### 2.1. DNA Methylation, Paternal Transmission, and Nutritional Influence

DNA methylation is crucial in the development and progression of cancer (Table 1), acting as an epigenetic modification that impacts gene expression and stabilizes the genome [28,29]. Aberrant methylations, such as hypermethylation of tumor suppressor genes and hypomethylation of oncogenes, are essential for the survival of abnormal cells and responsible for cell cycle, apoptosis, proliferation, drug resistance, metastasis, and intracellular signaling [30,31,32]. Paternal transmission of DNA methylation changes can influence cancer susceptibility in offspring, and the diet and body composition of fathers can modify male germline epigenetically, which affects the prospect of breast cancer in their daughters [33,34]. DNA methylation also plays a vital role in the growth and progression of ovarian cancer by affecting the expression of critical genes. Studies show that aberrant DNA methylation, characterized by global hypomethylation and region-specific hypermethylation, is common in tumor cells. Methylation changes can lead to chromosomal instability and inactivation of tumor suppressor genes, thereby contributing to ovarian cancer [35,36,37]. Methylation patterns of specific non-X-linked promoter CpG islands (CGIs) differ between tissues and their implications in normal development and cancer [38]. Some CGIs are heavily methylated in normal somatic tissues but unmethylated in germline cells, leading to gene silencing in somatic tissues while these genes are expressed in testis and sperm. These genes include *ANKRD30A*, *FLJ40201*, *INSL6*, *SOHLH2*, *FTMT*, *C12orf12*, and *DPPA* [38].

In cancer, these genes often lose their methylation and become abnormally expressed, with the extent of this hypomethylation varying among different genes in cancer cell lines [39,40]. DNA methyltransferase inhibitors can reactivate these silenced genes in cancer cells, and the hypomethylation and expression of these genes in cancer may trigger an immune response.

**Figure 1:**
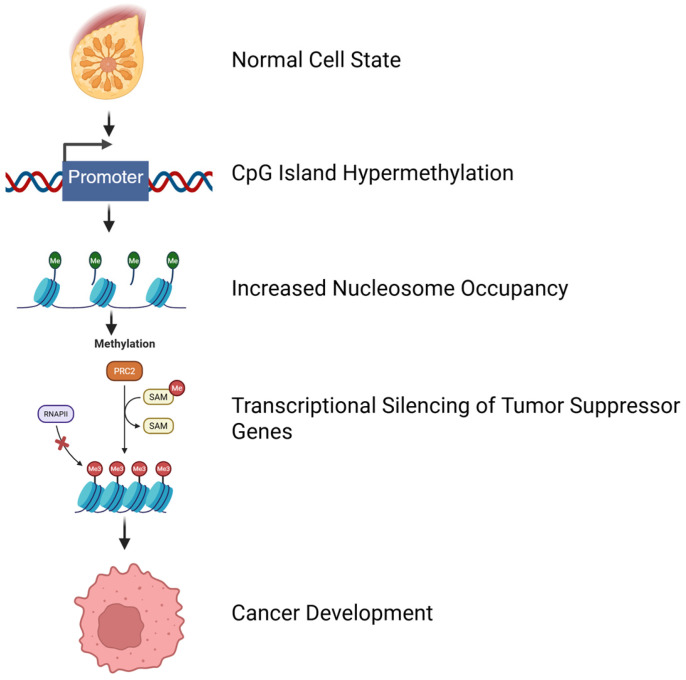
CpG island hypermethylation and cancer development. Promoter hypermethylation leads to increased nucleosome occupancy and silencing of tumor suppressor genes, driving cancer progression. Me is methyl group, SAM is S-adenosylmethionine, PRC2 is Polycomb Repressive Complex 2, and RNAPII is RNA Polymerase II. The bold arrow indicates the directional flow of the epigenetic changes leading to cancer development. Created in https://BioRender.com (accessed on 11 September 2025).

Additionally, other types of abnormal methylation in cancer include de novo methylation by DNMT3A/3B enzymes, and mutations in DNMT3B are associated with certain conditions [41,42]. DNA methylation plays a critical role in determining nucleosome occupancy, particularly in the 5′-CpG islands of tumor suppressor genes, demonstrating a bidirectional relationship where hypermethylated CpG islands are tightly linked to nucleosome presence [39,40]. Inducing DNA hypomethylation through genetic or pharmacological approaches leads to nucleosome eviction from previously hypermethylated CpG islands of tumor suppressor genes. This process is reversible, as demonstrated by the reoccupation of nucleosomes in de novo methylated CpG islands upon restoration of DNA methyltransferase activity [40,43]. Genomically, DNA hypermethylation and dense nucleosome occupancy consistently correlate with gene silencing, while hypomethylation and nucleosome eviction correspond to gene expression [40,44]. In cancer, promoter CpG island hypermethylation of tumor suppressor genes is a common epigenetic feature associated with transcriptional silencing (Figure 1), suggesting a link between hypermethylation of specific genes and cancer development [45,46].

**Table 1:** Role of DNA methylation and their functional consequences in cancer.

Type of Methylation	Function	Effect	Genes Affected	Reference
Hypomethylation	Occurs in tumor cells at the repetitive sequences residing in satellite regions	Chromosome breakage	Repetitive sequences in satellite or pericentromeric region	[47,48]
Hypomethylation	This leads to the activation of silenced genes and affects global DNA and specific genes	Gene activation	Silenced genes can activate proto-oncogenes and destabilize the genome promoting cancer progression	[47,49]
Hypermethylation	Often occurs at specific regulatory sites in the promoter regions or repetitive sequences	Tumor specificity	Genes involved in DNA repair and apoptosis, such as tumor suppressor genes	[50,51]
Hypermethylation	The heavy density of cytosine methylation in the CpG islands of the tumor suppressor gene promoters	Transcription block	Tumor suppressor genes	[50]
Aberrant Promoter Methylation	Leads to transcriptional silencing of tumor suppressors and metastasis inhibitor genes	Malignant and metastastic phenotype	Tumor suppressor	[51]

### 2.2. Histone Modifications in Paternal Transmission

Histone modifications (Table 2) have been implicated in carcinogenesis and also play a significant role in the regulation of gene expression and are primarily localized at the amino-terminal and carboxy-terminal tails of histones [52]. One of the extensively studied modifications is histone acetyltransferase (HATs), which catalyzes the addition of acetyl groups and promotes an “open” chromatin conformation, which means enhancing accessibility to transcriptional machinery. Histone deacetylases (HDACs) [53], which catalyze the removal of acetyl groups, result in chromatin compaction and transcriptional repression, in which, when aberrantly expressed, they can result in various hematological malignancies and solid tumors [54,55,56]. Furthermore, specific histone modifications can serve as a prognostic measure of cancer and the development of histone deacetylase (HDAC) inhibitors can alter the acetylation state of various proteins [57,58], such as histones, thereby influencing gene expression and potentially exerting anti-tumor effects [59,60,61]. Lower global levels of histone modifications have been correlated with more aggressive cancer subtypes in breast cancer [62,63]. The overexpression of HDAC2 is prevailing in breast cancer and may be a factor in the abnormal histone acetylation patterns discerned in malignancy [64]. Generally, reduced levels of histone modification such as H3K4me2 and H3K18ac are associated with defective prognosis across various types of cancer, including breast cancer [65,66,67]. A study emphasizes that alterations in histone modifications contribute to ovarian carcinogenesis by silencing tumor suppressor genes and facilitating the expression of oncogenes [68]. In ovarian cancer, histone modifications are being targeted to develop therapeutic interventions. Histone deacetylase inhibitors and other epigenetic therapies have shown potential in altering disease progression and improving outcomes when used in combination with other therapies [69,70]

Epigenetics changes in the germline, specifically in sperm, are now recognized as primary intermediaries in facilitating epigenetic transgenerational inheritance, which includes the transmission of environmentally induced epimutations to future generations without direct exposure [25]. In sperm, the active mark H3K4me3 and the repressive mark H3K27me3 have attracted significant attention. Research in mouse models reveal that overexpressing the histone demethylase KDM1A in the male germline can cause widespread loss of H3K4me3 at transcription start sites of developmental genes in sperm, leading to infertility, developmental abnormalities, and traits that persist for at least two subsequent generations [71,72]. Simultaneously, rodent studies show that while vinclozolin or DDT exposure usually leaves H3K27me3 levels largely unchanged, they prompt differential histone retention sites (DHRs) in the F3 generation, demonstrating that histone retention, rather than modification, may influence certain transgenerational contexts, with exposure-specific patterns [73,74]. Remarkably, H3K4me3 modifications in sperm bypass post-fertilization epigenetic reprogramming, remain in the early embryo, and act on genes involved in morphogenesis, metabolism, and pathways often dysregulated in cancer. Another contributing factor is the histone demethylase Kdm6a (Utx), a potential tumor suppressor, whose deletion in the paternal germline develop to extensive H3K27me3 redistribution, enhancer hypermethylation, and altered transcription factor binding in offspring somatic tissues, correlating with increased tumor incidence and reduced survival effects that are amplified by successive generations of Kdm6a loss [75].

**Table 2:** Major histone modification and their epigenetic roles.

Histone Modification	Description	Epigenetic Role	Location	Reference
H3 lysine 9 methylation (H3K9me3)	The addition of three methyl groups to the 9th lysine residue of histone H3	Associated with transcriptional silencing by recruiting HP1 (heterochromatin protein 1) to initiate and maintain heterochromatin formation	Constitutive heterochromatin	[76]
H3 Lysine 4 methylation (H3K4me3)	The addition of three methyl groups to the 4th lysine residue of histone H3	Associated with transcriptional activation and euchromatic regions	Promoter regions of active genes	[77]
H3 Lysine 27 methylation (H3K27me3)	The addition of three methyl groups to the 27th lysine residue of histone H3.	Associated with transcriptional repression particularly through Polycomb (Pc) group protein	Gene repressed by Polycomb group proteins	[78,79]
Histone acetylation	Histone acetyl transferases (HATs) add acetyl groups to histone tails	Facilitates gene transcription		[76]
Deacetylation	Histone deacetylases (HDACs) remove acetyl groups from histone tails	Inhibits gene transcription		[80,81]
Methylation	Histone methyltransferases add methyl groups to histone tails	Regulation of gene expression		[80,81]
Phosphorylation	Kinases add phosphate groups to histone tails	Regulation of gene expression, DNA repair, and chromosome condensation		[81]

### 2.3. Non-Coding RNAs (ncRNAs) in Paternal Transmission

The concept of paternal inheritance of disease risk via epigenetic imprinting is supported by several historical epidemiological studies of human cohorts from famine-affected regions worldwide. Such epigenetic imprints have been noted to be often sex-specific and pervasive through multiple generations [82,83]. During embryogenesis, parental DNA methylation marks undergo genome-wide reprogramming involving sequential erasure and re-establishment. While this reestablishment partially conserves parental methylation patterns, biparental inheritance and developmental programming generate a distinct methylomic landscape in the offspring that retains selective parental epigenetic signatures while establishing novel, lineage-specific methylation profiles [84].

This phenomenon necessitates investigation of epigenetic inheritance mediated by small non-coding RNAs (ncRNAs), including microRNAs (miRNAs), PIWI-interacting RNAs (piRNAs), tRNA-derived fragments (tRFs), long non-coding RNAs (lncRNAs), and similar regulatory RNA species that potentially escape global reprogramming and transmit epigenetic information across generations (Table 3). An emerging body of evidence supports the hypothesis that paternal exposure to environmental chemicals [85], psychosocial stressors [86], and dietary factors [87] directly influences epigenetic modifications in offspring, often at embryonic level via epigenetic reprogramming, potentially altering disease susceptibility, including cancer. Research in this area remains limited, with even fewer studies specifically investigating cancer-related mechanisms. In a 2018 study, da Cruz et al. demonstrated that low protein diet resulted in increased cancer risk in next generation female offsprings that is mainly mediated by aberrant modulation of small ncRNAs such as miR-28a, miR-92a, miR-200c, miR-451a, miR-191, miR-15b, and sperm-specific small RNA variants- tRF5-Gly-CCC, tRF5-Val-TAC [87]. Mounting evidence has shown that a vast majority (~60%) of protein coding mRNAs in humans have conserved seed sequence complementary to miRNAs [88], which are one of the common factors passed on to next generations through sperm. These ncRNAs are responsible for regulating expression levels of maternally contributed mRNA within a zygote [89]. Importantly, only a selected fraction of ncRNAs generated during spermatogenesis within the seminiferous tubules, and not the epididymis, becomes incorporated into the mature spermatozoon and subsequently transmitted to the next generation. This selective packaging occurs due to the highly specialized architecture of the sperm head, which undergoes extensive cytoplasmic reduction and chromatin compaction, limiting the retention of RNA species to those specifically associated with the condensed paternal genome or residual cytoplasm [85]. In a small cohort study conducted by Vaz et al. in 2021 [90], it was revealed that even a short-term change in the intake of micronutrients such as vitamin D, olive oil, and omega-3 fatty acids over 6 weeks can significantly modulate the expression of 112 piRNAs, 8 different categories of tRFs, and 15 miRNAs. These in turn dysregulate the expressions of genes downstream such as *ACAA2*, *ACSL1*, *CPT1A*, *ELOVL5*, *HADH*, *OXSM*, *PECR*, and *SCD* [90]. In other independent studies the genes *ACAA2*, *ACSL1*, *CPT1A*, *ELOVL5*, *HADH*, *OXSM*, *PECR*, and *SCD* have shown to sustain hallmarks of ovarian and breast cancer [91,92,93,94,95,96,97]. Such piRNAs include piR-004054, piR-004656, and piR001152, miRNAs include miR-513c-3p, mir-136-3p, and miR-4760-5ap, and tRFs include trf5b-TyrGTA, tir5-CysGCA, and trf5b-AlaAGC [91,92,93,94,95,96,97].

**Table 3:** Paternal microRNAs (miRNAs) related to breast cancer risk in vivo (mouse). Upward arrows indicate an increase, and downward arrows indicate a decrease. ** These genes have been found to be relevant in the context of stress response. These genes are also relevant to cancer progression. ‡ The corresponding cited study includes comprehensive catalog of ncRNAs relevant to cancer. Only a few relevant to cancer have been enlisted here.

Differentially Regulated Paternal miRNA	Expression	Target Pathway/mRNA	Cause	Organism	Reference
miR-28a, miR-92a, miR-200c, miR-451a, miR-191, and miR-15b	↑	AMP-activated protein kinase pathway (*Prkaa2*, *Cab39*), mammalian target of rapamycin (mTOR) signaling pathway	Paternal malnutrition, low protein diet	Mouse	[87]
miRNA-1896, miRNA-874 and miRNA-296-5p	↓	Hypoxia signaling, insulin receptor signaling, NANOG pathway, CDK5 signaling, epithelial–mesenchymal transition pathway, ERK/MAPK pathway, SAPK/JNK signaling, estrogen receptor signaling, April mediated signaling, axonal guidance signaling	Paternal obesity	Mouse	[33]
miR-29c, miR-30a, miR-30c, miR-32, miR-193-5p, miR-204, miR-375, miR-5323p, and miR-698	↑	*Sirt1*, *Ube3a*, *Srsf2*, *IL6st*, *Ncl*, *Aara*, *Agfg1*, *and Ralbp1* **	Chronic paternal stress	Mouse	[89]
miRNA- let-7d-5p, miR-10a-5p, miR-138-1-3p, miR-221-3p, miR-222-3p ‡			Exposure to toxicants (herbicide- Vinclozolin, fungicide, jet fuel, pesticide-DDT)	Rat	[85]
miR-30c, miR-30e, miR-124, miR-145, miR-361,miR-762 ‡	↑	Apoptosis, myogenesis, tumor suppression, immune response	Paternal exposure to radiation	Mouse	[98]
miR-29c, miR-134, miR-181a ‡	↓	Bax, Bcl, PTEN, stem cell survival			
Differentially regulated paternal tRF	Expression	Target pathway/mRNA	Cause	Organism	Reference
tRF-Gly-GCC, tRF-Gly-CCC, tRF-Val-CAC, tRF-Gly-TCC, tRF-Lys-CTT, and tRF-His-GTG	↑	Dub3, Ddr2, Tcstv3	Paternal low protein diet	Mouse	[99,100,101]
tRF5-Gly-CCC, tRF5-Val-TAC, tRF5-Pro-AGG and tRF5-Ser-CGA	↓	Wnt/β-catenin	Paternal low protein diet	Mouse	[87]
tRF5-Ile-TAT, tRF5-Arg-ACG, and tRF5-SeC-TCA	↑				
tRNA-Pro-AGG-1-2, tRNA-Pro-TGG-1-4, tRNA-Pro-AGG-1-M8	↓ (Potentially)	Trim7, Ccdc136	Exposure to toxicants (herbicide- Vinclozolin, fungicide, jet fuel, pesticide-DDT)	Rat	[85,102,103]

### 2.4. RNA Modification

Spermatozoa contribute not only to genetic DNA and retained histones but also to diverse modified RNAs, specifically small tRNA fragments (stRNAs) which are rich in chemical marks such as 5-methylcytidine (m5C) and N2-methylguanosine [21]. Beyond histone changes, DNA methylation, and small non-coding RNAs, RNA modification such as epitranscriptome represents a significant aspect of paternal epigenetic inheritance [21]. These modifications elevate RNA stability and modify their regulatory potential during the early stages of embryonic development. Study indicates that paternal nutritional stress, including diets high in fat or low in protein, alters both the quantity and modification profiles of sperm stRNAs. Strikingly, injecting stRNAs from diet-affected males into control zygotes can induce metabolic dysfunction in the F1 generation, showing a direct role for epitranscriptomic signals in paternal inheritance. Further insights from C. elegans reveal that sperm-specific paternal epigenetic inheritance (PEI) granules—containing Argonaute proteins like WAGO-3 bound to modified 22G-RNAs—are required for passing on epigenetically programmed responses across generations [104]. The conservation of PEI-like proteins (e.g., human BTBD7) suggests that comparable RNA-modification-dependent pathways may also exist in mammals [104].

Epitranscriptomic regulation play a vital role in gynecology-related cancer risk, especially in breast cancer, where the imbalance of RNA “writers” (e.g., METTL3), “readers” (e.g., YTHDF1/3), and “erasers” (e.g., FTO, ALKBH5) contribute to tumor development, cancer stem cell maintenance, metastasis, and resistance to treatment [105]. For example, overexpression of METTL3 enhances cell proliferation and resistance to chemotherapy, while ALKBH5-driven demethylation of pluripotency genes promotes breast cancer stemness. Remarkably, FTO, an m6A/m6Am RNA demethylase initially identified through its association with obesity, is associated with breast cancer-related SNPs and mechanistically connects metabolic status with cancer susceptibility [105]. Since YTHDC2 and ALKBH5 also regulate spermatogenesis, m6A modification is positioned at the crossroad of germline integrity, fertility, and transgenerational epigenetic communication. Dietary exposures like high-fat diets, miRNAs from bovine milk exosomes, and altered glucose metabolism can affect the expression or activity of epitranscriptomic regulators such as METTL3 and FTO [105,106]. This creates a conceptual framework in which paternal diet-induced RNA-modification changes in sperm and obesity-linked epitranscriptomic dysregulation in women coincide on shared RNA-based mechanisms that influence reproductive health and gynecological cancer risk [107].

### 2.5. Epigenetics Regulations in Breast and Ovarian Cancer

**Figure 2:**
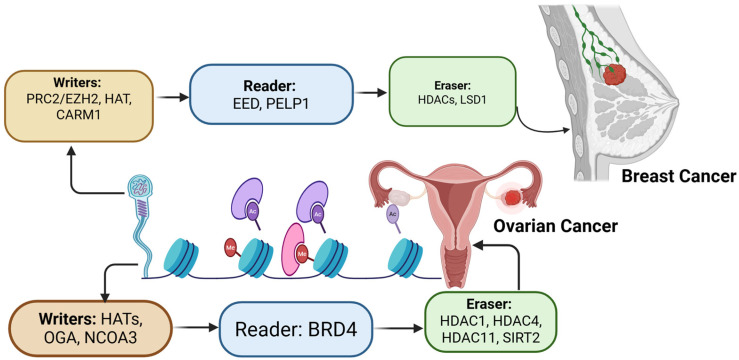
Altered histone codes in ovarian and breast cancer. Epigenetic regulation of chromatin involves three classes of protein. Writers (protein that add modifications), readers (proteins that recognize and interpret these modifications), and erasers (enzymes that remove them). Dysregulation of these enzymes alters gene expression and contributes to breast and ovarian cancer. The arrows indicate the sequential progression of the epigenetic regulations. Created in https://BioRender.com (accessed on 11 September 2025).

In ovarian cancer (OC), the disruption of histone acetylation—regulated by the balance between histone acetyltransferases (HATs) and histone deacetylases (HDACs)—is significant, with 37 out of 40 acetylation-related genes showing different expression levels in OC compared to normal tissues. An eight-gene histone acetylation signature (SIRT5, BRD4, OGA, SIRT2, HDAC4, NCOA3, HDAC1, and HDAC11) has been identified as an independent prognostic marker, where seven genes are linked to poorer outcomes, while SIRT5 is associated with a favorable prognosis [108]. These changes affect pathways like Wnt signaling, leading to platinum resistance and reduced anti-tumor immunity. Specific disrupted “histone codes” in OC include OGA, an O-GlcNAcase that modulates histone acetylation indirectly via O-GlcNAcylation crosstalk (influences p53 stability), NCOA3 (a co-activator linked to platinum resistance), BRD4 (a reader that enhances transcription and can be targeted by inhibitors), and erasers such as SIRT2, HDAC1, HDAC4, and HDAC11, whose altered activities influence drug sensitivity, tumor progression, and survival [108]. Similar disruptions occur in breast cancer, where NCOA3, OGA, HDAC1, and HDAC11 exhibit cancer-type-specific prognostic effects. Dietary compounds such as curcumin have been shown to affect histone acetylation [109]. Curcumin, a component found in turmeric, exerts protective effects against cancer by activating tumor suppressor genes and inhibiting oncogenes [109]. Overall, these findings underscore that abnormal histone acetylation and methylation in germline and somatic cells not only contribute to ovarian cancer pathogenesis but may also serve as inheritable risk factors and actionable therapeutic targets [108]. H3K4me2 and H3K27me3 play a part in transgenerational inheritance by preserving the developmental gene expression state in sperm [110,111]. This involves Trithorax MLL family and Polycomb (PRC2/EZH2) functioning as writers, EED acting as a reader, and LSD1/KDM1 as an eraser. Dysregulation of these marks can elevate the risk of cancer in offspring [112]. In breast cancer, altered histone codes are essential to tumor initiation, progression, resistance to therapy, and metastasis. Writers (HATs, HMTs like CARM1, and the MLL family) add activating or repressive marks, readers (e.g., PELP1 recognizing H3R17me2a/H3R26me2a) interpret these modifications and recruit co-regulators, and erasers (HDACs, KDM1/LSD1, HDAC3) remove marks to silence or reprogram transcriptional states [113,114] (Figure 2). Writers, readers, and erasers not only contribute to tumorigenesis within an individual but may also enable the heritage transmission of oncogenic across generations [115].

## 3. Nutrition Impacts on Sperm Epigenetics and Offspring’s Gynecological Cancers

Recent studies demonstrate that specific bioactive food compounds derived from obesogenic diets can cause stable yet dynamic epigenetics modifications in sperm which in turn affect offspring health and disease susceptibility. Epigenetic modifications such as DNA methylation, histone modification, and small non-coding RNAs are heritable through fertilization thereby supporting the Paternal Origins of Health and Disease (POHaD). Paternal undernutrition and overnutrition have been linked to breast cancer risk in daughters [116], and paternal obesity has been connected with enhanced susceptibility to breast cancer in daughters. Paternal malnutrition has also resulted in a greater occurrence of mammary cancer in female offspring, with these tumors materializing earlier and growing more rapidly compared to controls [87]. Emanated evidence shows that comparing unhealthy fast foods (e.g., fries, pizza) with nutrient dense diets like fruit, nuts, whole grains, and vegetables convey contrasting sperm epigenetic patterns. Regular ingestion of fast food is linked to altered methylation at imprinted gene loci (e.g., IGF2, MEG3-IG), lower sperm motility (directionality of methylation change vary by locus), and increased risk of transferring adverse metabolic phenotype to offspring [117]. Implied mechanisms include oxidative stress from high fat and carbohydrates, acrylamide exposure including DNA damage, trans-fatty acid accumulation impairing Sertoli cell lipid metabolism, and endocrine-disrupting chemicals like BPA, phthalates altering sperm methylation [117]. These paternal epigenetic alterations are connected to offspring predisposition to chronic metabolic disorders, cancer, obesity, diabetes, and cardiovascular dysfunction, even across multiple generations. Contrarily, consuming vegetables, whole grains, fruits, nuts, and polyunsaturated fatty acids is associated with positive sperm outcomes, such as reduced methylation at NNAT, IGF2, and MEG3 loci, higher total motile count, and improved semen volume [117,118]. Such provident diets amplify sperm epigenetic integrity, enhance reproductive success, and boost antioxidant defenses. This protective effect extends to offspring, leading to lower risk of metabolic dysfunction and better fetal growth [118].

Micronutrients and B vitamins, specifically folate, play a crucial role through one carbon metabolism and methyl group donation [119]. Folate deficiency in fathers alters sperm methylation and histone marks (H3K4, H3K9), leading to developmental abnormalities in offspring, placental defects, increased pregnancy loss, and a higher risk of chronic diseases such as cancer and diabetes [119,120]. Human studies also show that disruptions in folate metabolism, particularly due to MTHFR polymorphisms, are linked with idiopathic male infertility and abnormal sperm methylation [118,119]. Importantly, both folate deficiency and excessive supplementation can negatively influence the sperm epigenome and offspring outcomes, emphasizing the importance of balanced intake [118]. Animal and human studies also underline the effects of high fat diets and high sugar diets. High fat diets induced abnormal DNA methylation at imprinted genes, histone mark changes (H3K27me3), and altered sperm small ncRNAs such as miRNA, tsRNA, piRNAs which are transmitted to offspring, leading to glucose intolerance, insulin resistance, hypoandrogenism, obesity, and pro-inflammatory phenotypes across F1 and F2 generations. Similarly, short-term high sugar diet (HSD) alters sperm small non-coding RNA (sncRNA) composition, particularly mitochondria tsRNAs, linking paternal dietary sugar intake with sperm motility, oxidative stress, and potentially altered embryonic gene regulation [118,119,120].

### 3.1. Macronutrient on Sperm Epigenetics

**Figure 3:**
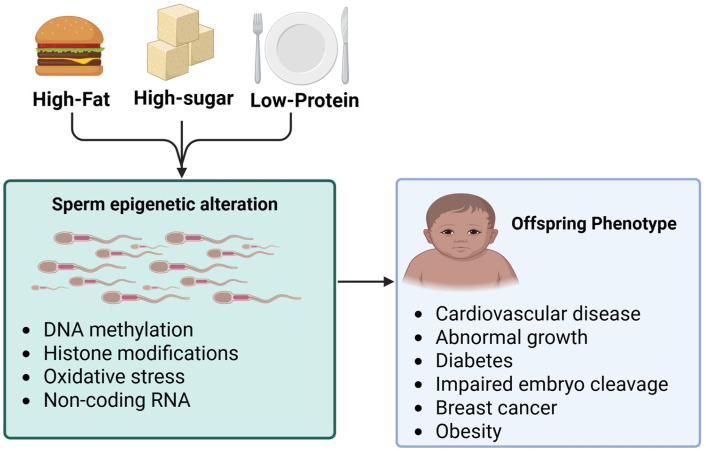
Paternal macronutrient imbalance such high-fat diet, high-sugar, and low protein diets induce changes in sperm through epigenetic mechanisms. Altered DNA methylation, histone modification, and small ncRNAs signals reprogram early embryonic development and pass to F1–F2, leading to metabolic dysfunction, cancer, and developmental effects. The bold arrow represents the directional flow from paternal diet sperm epigenetic alteration. Created in https://BioRender.com (accessed on 11 September 2025).

Imbalanced paternal macronutrient intake such as diets high in fat and high sugar Western diet, low in protein, or excessive high protein processed meats (bodybuilder foods), along with high caloric intake and early dietary exposures play a vital role in influencing sperm epigenetics and offspring health (Figure 3). Studies show that dietary imbalances disrupt sperm through epigenetic mechanisms, impacting not only fertility but also the developmental trajectory and long-term disease risk of descendants. High sugar diet impairs sperm motility and fertilization, contributes to adverse embryonic and offspring phenotypes, and intensifies oxidative stress [121], which works in hand with high fat diet [121]. Hyperglycemia and high-glycemic load exposure aggravate ROS and epigenetic instability, linking diet-induced glucose dysregulation to male infertility and intergenerational metabolic issues [121]. Similarly, high fat diet consistently induces significant changes in sperm DNA methylation, including global hypomethylation and imprinting errors (notably at IGF2 DMRs—Differentially Methylated Region) [122], also affecting histone acetylation and the exchange of histones for protamine, leading to less condensed chromatin that is susceptible to oxidative damage. These diets alter small RNA content, tsRNAs, miRNAs, lncRNA, and RNA modifications such as m6A. Paternal high fat diets increase reactive oxygen species (ROS) and DNA fragmentation [123], alter the composition and microbiota of seminal plasma, and disrupt the blood–testis barrier [122,123]. The consequences range from reduced sperm motility [123] and viability to delayed embryo development, fewer blastocyst cells, and implantation failure [123]. In offspring, metabolic syndrome traits such as insulin resistance, β-cell dysfunction, and obesity manifest alongside reproductive subfertility, neurobehavioral changes like increased anxiety and impaired learning, cardiovascular disease, and elevated cancer susceptibility. Many of these outcomes persist into the F2 generation, supporting the concept of “inherited metabolic memory [124].”.

High protein diets dominated by processed red meat (e.g., bodybuilder meals) indirectly play a part in infertility by reducing sperm count, testicular volume, testosterone, and motility. Mechanisms likely involve endocrine disruption from fat residues, preservatives, and hormone contaminants, along with increased oxidative stress. Additionally, low protein diets affect genome-wide sperm DNA hypomethylation and disrupt one carbon metabolism, downregulating Dnmt1, Dnmt3L, and folate-cycle enzymes [123]. They alter sperm and seminal plasma tRNA content, impair spermatogonia stem cell populations, and lower serum testosterone. Through altered seminal plasma signaling, low protein diets influence uterine gene expression and early embryonic development, resulting in cardiometabolic dysfunction, hypertension [123], vascular abnormalities, smaller fetuses, stalled blastocyst development, and altered lipid metabolism in offspring [125]. Many of these effects are dependent, with male offspring particularly vulnerable, and some persist into the F2 generation.

### 3.2. Micronutrients and Sperm Epigenetics

Sufficient levels of micronutrients are increasingly recognized as critical for preserving the epigenetic integrity of sperm [126]. Deficiency in folate and other B vitamins such as B2, B6, and B12 as well as minerals like iron [119,126], magnesium, iodine, selenium, manganese, and zinc have been shown to affect paternal germline programming [127]. One-carbon vitamins are crucial for regulating the availability of S-adenosylmethionine (SAM), a universal methyl donor essential for DNA and histone methylation, meaning that a lack of folate or B12 deficiency can obstruct the methylation imprinted genes, global methylation balance, and spermatogenesis [128]. Animal studies have shown that paternal folate deficiency can alter sperm methylation patterns, reduce sperm count, and impact offspring with developmental issues such as growth restriction and higher metabolic risk [129]. Additionally, both low and excessively high folic acid intake have been associated with changes in seminal DNA methylation and altered embryonic growth trajectories in human studies [130,131]. Roles of these vitamins in sperm are shown in Table 4. Minerals influence various pathways, including chromatin stability, oxidative stress, antioxidant defense, and endocrine regulation. Calcium, magnesium, phosphorus, zinc, iron, and potassium are indispensable for spermatogenesis and sperm function. Potassium, alongside sodium, maintains osmotic balance and regulates ion channels critical for sperm motility and hyperpolarization, with imbalances impairing spermatogenesis [127]. Phosphorus, a fundamental component of nucleotides (AMP/ADP/ATP), drives energy transfer and supports DNA/RNA structure, linking deficiency to reduced fertility [127]. Calcium regulates capacitation, motility, and acrosome reaction, with both deficiency and excess impairing fertilization competence and increasing oxidative stress. Zinc, abundant in seminal plasma, aids in protamine cross-linking, antioxidant defense [132], and chromatin packaging, and deficiency in zinc can lead to low sperm count and motility, chromatin destabilization, and increased oxidative damage [129]. Notably, zinc supplementation can restore sperm DNA integrity and reduce negative metabolic programming in offspring, as observed in rodent models. Additionally, selenium is crucial for the activity of selenoproteins, such as glutathione peroxidase, which protects sperm DNA from reactive oxygen species (ROS) [132]. Importantly, a lack of selenium in fathers has been shown to alter mammary gland development and increase breast cancer risk in female offspring, thereby providing an example of a direct link between micronutrients, epigenetics, and cancer risk in offspring.

The significant limitations that are evident throughout all the references in Table 4 are that they lack well-controlled human trials and difficulties in applying results from animal studies to humans’ reproductive biology. Short-term interventions might enhance specific sperm or embryo parameters but often do not address the underlying metabolic dysfunction. Additionally, current genetic analyses often ignore compound allele interaction, and the ideal nutrient concentrations and supplementation thresholds are yet to be determined.

### 3.3. Influence of Paternal Obesity on Female Reproductive Health

**Figure 4:**
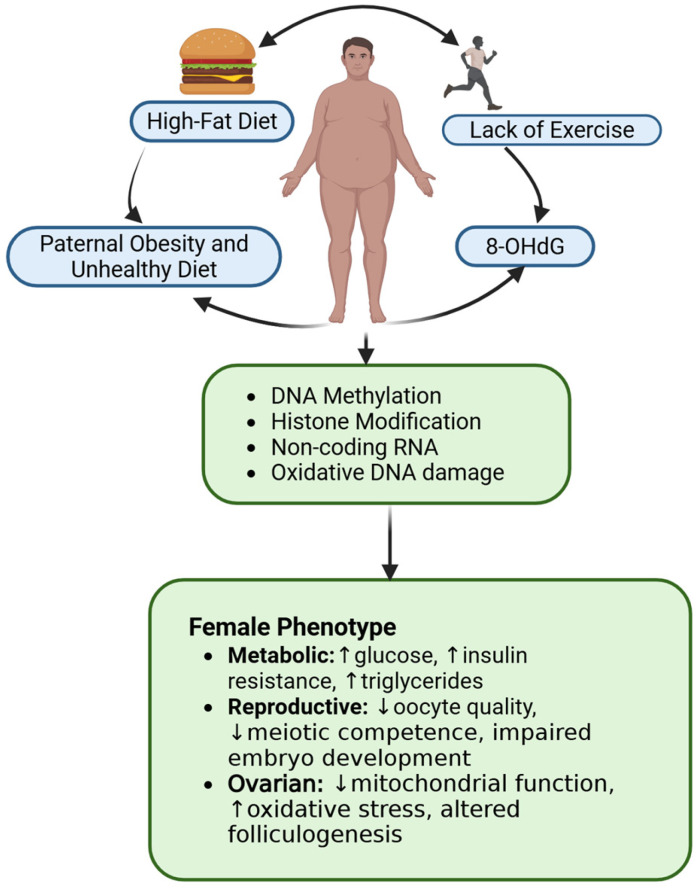
Mechanistic schema on how paternal obesity influences female reproductive health. These molecular alterations are passed on during fertilization, leading to compromised early embryonic development such as delayed cell cycles, and subsequent intergenerational health risks. 8-OHdG is 8-hydroxy-2-deoxyguanosine, an oxidized form of DNA that is biomarker for oxidative stress and DNA damage. Upward arrows indicate an increase, and downward arrows indicate a decrease or low in the female phenotype. The arrow outside the box indicates directional flow of the figure. Created in https://BioRender.com (accessed on 11 September 2025).

Paternal nutrition, especially when it leads to obesity, has significant implications for female infertility and the broader reproductive health of future generations (Figure 4). Increasing evidence has highlighted the role of paternal obesity in influencing offspring health, not only through genetic inheritance but also via epigenetic and environmental factors [136]. Obesity can alter sperm quality through different pathways such as hormonal imbalances, oxidative stress, and epigenetic modifications [137]. These factors can lead to low sperm viability [138] and DNA damage, contributing to developmental issues in offspring and indirectly affecting female fertility [130,137]. Reduced sperm quality due to obesity can impair fertilization processes, leading to suboptimal embryonic development and higher miscarriage rates [139,140]. Animal studies have shown that paternal obesity, often induced by a high-fat diet (HFD), affects the reproductive health of at least two subsequent generations. In mice, obesity in fathers led to diminished reproductive functions in both male and female offspring over two generations, suggesting a transgenerational impact of paternal metabolic state on fertility [141]. The offspring of obese fathers exhibit metabolic and reproductive challenges, highlighting the importance of paternal health at the time of conception [141]. Additionally, paternal obesity can cause endocrine disruptions that alter luteinizing hormone and testosterone levels, intensifying reproductive difficulties in female offspring when exposed to a similar high-fat environment after birth [142], obesity-related hormonal imbalance, such as hypogonadotropic hypogonadism [142], and elevated pro-inflammatory markers in semen, including IL-8 [137], may further impair the hypothalamic–pituitary–ovarian axis, a critical regulator of female reproductive health. In addition, obesity-associated changes in seminal fluid composition and sperm DNA methylation can further compromise reproductive outcomes [142]. Studies in rodents reveal that a father’s metabolic condition can reprogram the zygote by altering sperm DNA methylation, histone modifications, and small non-coding RNAs (miRNAs, tsRNAs) [117,143], along with causing oxidative DNA damage [144], hence influencing developmental pathways [145,146]. In female offspring (F1), these paternal influences result in impaired oocyte quality [147], diminished meiotic competence, elevated ROS, delayed embryo development (at the 2- and 8-cell stages and caused lower blastocyst quality) [130,147]. Additional alterations include disrupted ovarian metabolism-characterized by increased lipid accumulation and GLUT4-expression in cumulus-oocyte complexes-and mitochondria/redox imbalances such as reduced mtDNA copy number, downregulated PGC-1α/TFAM/NRF1 and AMPK [148], and diminished TAC and Nfe2l2/NRF2 activity, alongside decreased miR-149 expression [130].

Exercise, nutrition, and lifestyle changes have been proposed to mitigate the negative effects of paternal obesity. For instance, engaging in exercise prior to conception has shown potential in improving sperm quality and, subsequently, offspring health [130]. Therefore, addressing paternal obesity through nutrition management may enhance reproductive outcome and aid in managing infertility issues not only in women but also in future generations.

### 3.4. Phytochemicals Influence Sperm Epigenetics

**Figure 5:**
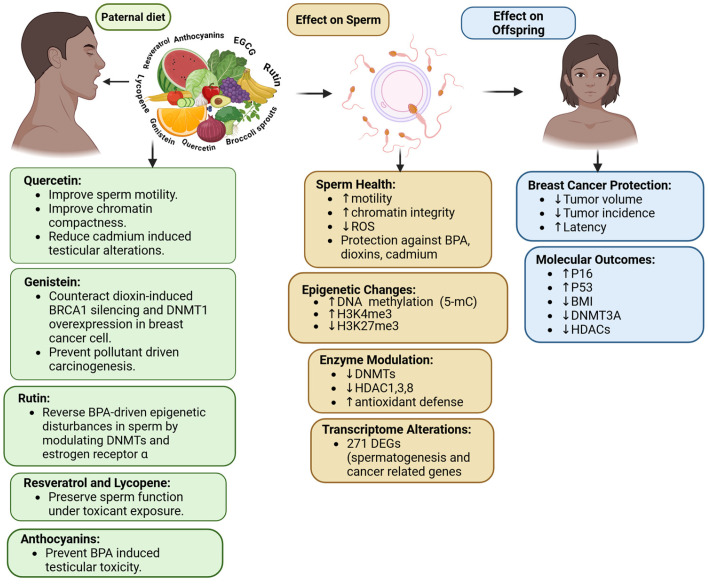
Paternal intake of phytochemical influence on sperm epigenetics. This includes changes in DNA methylation, histone modifications, enzyme activity, transcriptome, and sperm quality. These germline changes contribute to a reduced risk of breast cancer in their offspring by decreasing the incidence and tumor volume, delaying latency, and modulating genes associated with tumors. Upward arrows indicate an increase, and downward arrows indicate a decrease in the outcome. Created in https://BioRender.com (accessed on 11 September 2025).

Dietary phytochemicals consumed by fathers, particularly bioactive compounds derived from plants like sulforaphane found in broccoli sprouts (SFN), epigallocatechin-3-gallate (EGCG) from green tea polyphenols, carotenoids, flavonoids, and resveratrol influentially impact sperm health (Figure 5), epigenetic programming, and the cancer susceptibility of their offspring [149,150]. Research has shown that when the father consumes SFN-rich broccoli sprouts and EGCG-rich green tea polyphenols, there is significant suppression of estrogen receptor-negative (ER-) mammary tumor development in female offspring, as seen in transgenic mouse models [149]. This protective effect is synergistic when these compounds are combined, leading to a lower tumor incidence, smaller tumor size, and delayed tumor latency [141]. These effects are driven by germline epigenetic inheritance, where the paternal diet alters sperm molecular profiles, transmitting these changes to the next generation. In sperm, the presence of SFN and EGCG from the paternal diet altered the transcriptome, with 271 genes showing differential expression, including those linked to spermatogenesis and breast cancer progression, and 467 differentially methylated regions (DMRs) associated with cancer-related pathways. Notably, changes in sperm DNA methylation alone did not fully account for gene expression regulation, suggesting a complex interaction of multiple epigenetic markers [150,151]. These modifications were mirrored in the mammary tumors of offspring, where paternal phytochemicals reduced HDAC enzymatic activity, elevated global H3K4 methylation, decreased H3K27 methylation, and increased global DNA 5-methylcytosine levels [149]. Tumor suppressor proteins (p16, p53) were upregulated, while oncogenic HDACs (HDAC1, HDAC3, HDAC8) and BMI1 were downregulated [149].

The Mediterranean diet is a model “epigenetic diet,” rich in whole grains, vegetables, fruits, legumes, olive oil, fish, and polyphenols, with proven inverse associations with cancer, cardiovascular, and metabolic diseases. Beyond SFN and EGCG, nutraceutical components such as curcumin, lycopene, quercetin, and resveratrol modulate DNA methylation, miRNA expression [150], and histone acetylation, functioning as neutral epigenetic regulators that suppress oncogenes, reactivate tumor suppressor genes, and attenuate inflammation and oxidative stress [152]. Additionally, it has been shown that quercetin, rutin, genistein, luteolin, apigenin, hesperetin, morin, daidzein, anthocyanidins, and resveratrol have positive effects on male reproductive health and sperm integrity, specifically under environmental pollutant stress [152]. These compounds exert their effects through antioxidants, chelation, anti-inflammatory, and epigenetic pathways. Epidemiological evidence demonstrates that adherence to the Mediterranean diet reduces cancer mortality and incidence. Paternal diet can also counteract the transgenerational carcinogenic risks posed by endocrine disruptors and pollutants, thereby preserving male fertility and potentially reducing cancer risk in offspring [150,151,152].

## 4. Conclusions

The review presented here indicates that paternal epigenetic inheritance plays a critical, though often overlooked, role in influencing breast and ovarian cancer risk in offspring. The paternal diet, particularly intake of macro- and micronutrients as well as bioactive compounds, can induce epigenetic changes in sperm DNA methylation, histone modifications, and non-coding RNAs. These alterations in sperm are capable of bypassing reprogramming during embryonic development and influence gene expression patterns in female offspring, potentially modulating cancer susceptibility. Key findings include the impact of paternal folate deficiency on sperm DNA methylation, the role of paternal nutrition in increasing breast cancer risk in daughters, and the protective effects of paternal intake of phytochemicals like sulforaphane against mammary tumors in offspring. Overall, this review underscores the need to consider paternal nutrition as an important factor in female cancer prevention strategies.

## 5. Limitation and Future Directions

One major limitation of the current research in this field is the reliance on animal models, particularly rodents, for many of the mechanistic studies. While these models provide valuable insights, the translation of findings to humans requires caution due to differences in reproductive biology and epigenetic reprogramming between species. Additionally, most human studies are observational and cannot establish causality. The long-term nature of transgenerational effects also poses challenges for conducting controlled human studies. Furthermore, while maternal factors have been extensively studied, the impact of paternal lifestyle and nutrition in modulating gynecological cancer susceptibility across generations is an emerging area that requires further exploration.

Future research should focus on validating findings from animal models in human cohorts through long-term epidemiological studies and clinical trials. Mechanistic studies are needed to show how specific dietary components influence sperm epigenetic marks and how these are maintained during embryonic reprogramming. Advanced sequencing technologies should be employed to comprehensively profile the sperm epigenome and identify robust biomarkers of paternal dietary exposures. The potential for dietary interventions in men to reduce gynecological cancer risk in offspring warrants further investigation. Additionally, the interaction between paternal and maternal dietary factors in modulating offspring cancer risk should be explored. Finally, the development of non-invasive methods to assess sperm epigenetic profiles could facilitate larger-scale human studies and potentially inform personalized preconception care strategies aimed at reducing breast and ovarian cancer risk in future generations.

## Figures and Tables

**Table 4:** Impact of micronutrient deficiencies on sperm epigenetics and offspring health.

Micronutrient	Role in Sperm	Deficiency Effects on Sperm	Offspring/Health Consequences	Organism	References
Folate (B9)	One-carbon metabolism, DNA and histone methylation, nucleotide synthesis	Low sperm count, increase DNA damage	Infertility, congenital malformations	Mice, rat	[119]
Vitamin B6/B12	DNA synthesis, cofactors in homocysteine metabolism	Chromosomal instability, hypomethylation	Altered DNA methylation	Human, mice, rat	[133,134]
Vitamin C	Testosterone regulation, antioxidant protection	Low motility and sperm count, DNA oxidation	Protects against smoking-related sperm DNA damage	Human, mice, rat	[132]
Vitamin D	Vitamin D receptor in sperm/testis, calcium transfer	Altered morphology, low sperm count	Infertility, interacts with epigenetic regulation	Human, mice, rat	[132]
Vitamin E	Protects DNA from ROS, antioxidant	Oxidative DNA damage		Human, mice, rat	[132]
Iron (Fe)	Integral to Heme proteins and support DNA/RNA structure	Impaired Spermatogenesis	Developmental and metabolic risk	Rat	[127]
Iodine	Thyroid-dependent spermatogenesis	Testicular atrophy with hypothyroxinemia, decrease motility and sperm count		Rat, goat	[127]
Zinc	Chromatin stability, protamine cross-linking; antioxidant enzymes; transcriptional cofactors	Oxidative damage, decrease motility/morphology, poor chromatin integrity	Offspring cancer risk through germline DNA damage	Rat	[134,135]
Selenium	Sperm maturation, Selonoproteins (GPx-1 cofactor)	ROS accumulation, reduces motility	Altered mammary development and increases breast cancer risk in daughters	Mice	[135]
Magnesium	Glutathione synthesis	Oxidative DNA damage, energy dysregulation		Rat, goat	[127]

## Data Availability

No new data were created or analyzed in this study.

## Abbreviations

ANKRD30A	Ankyrin Repeat Domain 30
ACAA2	Acetyl-CoA Acyltransferase
ACSL1	Acyl-CoA Synthetase Long Chain Family Member 1
Agfg1	ArfGAP with FG repeats 1
AMPK	Adenosine Monophosphate-activated Protein Kinase
*BRCA1* and *BRCA2*	Breast Cancer Susceptibility gene
Bax	BCL2-Associated X
Bcl	B Cell Lymphoma Protein
BMI1	Polycomb Ring Finger 1
BPA	Bisphenol A
BRD4	Bromodomain Containing 4
Ccdc136	Coiled-Coil Domain Containing 136
CARM1	Coactivator-Associated Arginine Methyltransferase 1
CpG	Cytosine Phosphate Guanine
CGI	CpG Island
CDK5	Cyclin-Dependent Kinase 5
CPT1A	Carnitine Palmitoyltransferase 1A
Cab39	Calcium Binding Protein 39
C12orf12	Coiled-coil Glutamate Rich Protein 1
DDT	Dichlorodiphenyltrichloroethane
DHRs	Differential Histone retention
DMR	Differentially Methylated Regions
DPPA	Developmental Pluripotency Associated gene
DNMT	DNA Methyltransferase protein
EGCG	Epigallocatechin Gallate
ELOVL5	Fatty Acid Elongase 5
ERK	Extracellular Regulated Kinase
ER	Estrogen Receptor
EZH2	Enhancer of Zeste Homolog 2
FLJ40201	Ciliary Microtubule-associated Protein 2
*FTMT*	Ferritin Mitochondrial gene
GLUT4	Glucose Transporter 4
GPx-1	Glutathione Peroxidase 1
HER2	Human Epidermal Growth Factor 2
HADH	3-hydroxyacyl-CoA Dehydrogenase
HAT	Histone Acetyltransferase
HMT	Histone Acetyltransferase
HDAC	Histone Deacetylase
H3K4me2	Histone 3 lysine 4 dimethylation
H3K18ac	Histone 3 lysine 18 acetylation
H3K27me3	Histone 3 lysine 27 trimethylation
H3K9me	Histone 3 lysine 9 methylation
HFD	High Fat Diet
IGF2	Insulin-like Growth Factor 2
*INSL6*	Insulin Like 6 gene
IL6st	Glycoprotein 130 (includes Interleukin 6)
IL8	Interleukin 8
JNK	c-Jun N-terminal Kinase Family Protein
KDM1/LSD1	Lysine-specific Demethylase 1
Kdm6a	Lysine-specific Demethylase 6A
lncRNA	Long non-coding RNAs
miRNAs	MicroRNA
mTOR	Mammalian Target of Rapamycin
MAPK	Mitogen Activated Kinase-like Protein
MEG3	Maternally Expressed 3 (long non-coding RNA)
*MEG3-IG*	Maternally Expressed Gene 3-intergenic Differentially Methylated Region
m6A	N6-methyladenosine
MLL	Mixed-lineage Leukemia
MTHFR	Methylenetetrahydrofolate Reductase
NANOG	Homeobox Transcription Factor
NNAT	Neuronatin
Ncl	Nucleolin
NCOA3	Nuclear Receptor Coactivator 3
NRF1	Nuclear Respiratory Factor 1
Nfe2l2	Nuclear Factor Erythroid 2-related Factor 2
ncRNA	Non-coding RNA
OGA	O-GlcNAcase protein
OXSM	3-oxoacyl-ACP Synthase
PRC2	Polycomb Repressive Complex 2
piRNA	PIWI-interacting
PECR	Peroxisomal Trans-2-Enoyl-CoA Reductase
PTEN	Phosphatase and Tensin Homolog
PI3K	Phosphoinositide 3-Kinase
PR	Progesterone Receptor
PR+	Progesterone Receptor-Positive
Prkaa2	Alpha-2 Catalytic Subunit of AMP-activated Protein Kinase
PELP1	Proline-, Glutamic acid-, and Leucine-rich Protein 1
PGC-1α	Peroxisome Proliferator-activated Receptor-gamma Coactivator 1-alpha
POHaD	Paternal Origins of Health and Disease
RNAPII	RNA Polymerase II
Ralbp1	RalA Binding Protein 1
ROS	Reactive Oxygen Species
SOHLH2	Spermatogenesis and oogenesis specific basic helix-loop-helix 2
SAM	S-adenosylmethionin
SAPK	Stress-Activated Protein Kinase
SCD	Stearoyl-CoA Desaturase
Sirt	Sirtuin
SFN	Sulforaphane
Srsf2	Serine and arginine rich splicing factor 2
TFAM	Mitochondrial Transcription Factor A
tRFs	tRNA-derived fragments
tsRNA	tRNA-derived small RNAs
tRNA	Transfer RNA
TAC	Total Antioxidant Capacity
Trim7	Tripartite Motif Containing 7
TMC	Total Motile Count
Ube3a	Ubiquitin Protein Ligase E3A (E6-AP) Protein
Utx	Ubiquitously Transcribed Tetratricopeptide Repeat on Chromosome X
Wnt	Wingless-related integration site
